# Fatty Acid Binding Protein 4 Regulates the Antigen‐Presenting Function of Dendritic Cells Resulting in T Cell Priming in Streptozotocin‐Induced Type 1 Diabetes Mice

**DOI:** 10.1111/1753-0407.70123

**Published:** 2025-07-26

**Authors:** Hailan Zou, Lingxiang Xie, Jingyi Hu, Rong Zhang, Yanfei Wang, Aimin Xu, Zhiguang Zhou, Xiaoyu Xiao, Yang Xiao

**Affiliations:** ^1^ National Clinical Research Centre for Metabolic Diseases, Key Laboratory of Diabetes Immunology, Ministry of Education, and Department of Metabolism and Endocrinology The Second Xiangya Hospital of Central South University Changsha Hunan China; ^2^ The First People's Hospital of Foshan, Department of Endocrinology Sun Yat‐Sen University Foshan Guangzhou China; ^3^ State Key Laboratory of Pharmaceutical Biotechnology, Department of Medicine, and Department of Pharmacology & Pharmacy The University of Hong Kong Hong Kong China; ^4^ Department of Nutrition Xiangya Hospital, Central South University Changsha Hunan China

**Keywords:** autoimmunity, dendritic cells, fatty acid binding protein 4, type 1 diabetes

## Abstract

**Background:**

Type 1 diabetes is an autoimmune disease with progressive destruction of insulin‐producing β cells in islets of Langerhans of the pancreas. However, the early pathogenic factors triggering the recruitment and activation of innate immune cells remain unclear. A study reported that FABP4 accelerates the onset of type 1 diabetes in NOD mice by inducing the polarization of proinflammatory macrophages and their infiltration into pancreatic islets. Nonetheless, the role of FABP4 in mediating crosstalk between innate immunity and adaptive immunity in T1D is unclear.

**Methods:**

Intraperitoneal injections of streptozotocin were used to establish a type 1 diabetes mouse model. Blood glucose was monitored, and intraperitoneal glucose tolerance test (IPGTT) was conducted to compare glucose homeostasis. The peripheral immune cells were detected using flow cytometry. Mixed lymphocyte reactions were applied to examine the function of FABP4 on antigen‐presenting in dendritic cells.

**Results:**

We found that genetic ablation of *FABP4* in mice alleviated STZ‐induced diabetic damage by reducing diabetogenic T lymphocytes and their production of inflammatory cytokines. In vitro studies, *FABP4* deficiency dendritic cells expressed lower properties of CD86 and CD80, showing impaired antigen‐presenting functions.

**Conclusions:**

Genetic ablation of FABP4 in mice alleviated STZ‐induced diabetic damage by impairing the antigen‐presenting function of dendritic cells through downregulating the phosphorylation levels of the ERK and JNK pathways.

## Graphical abstract

FABP4 deficiency alleviates the STZ‐induced autoimmune destruction of pancreatic β cells in mice. Mechanistically, FABP4 facilitates DC‐mediated T lymphocyte proliferation, inflammatory cytokine production, and naïve CD4^+^ T cell differentiation by promoting the activation of DCs and enhancing the antigen‐presenting capacity of DCs.

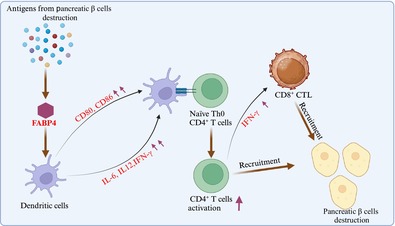

**Summary**

- FABP4 deficiency alleviates the STZ‐induced T1D phenotype in mice by reducing the proportion of diabetogenic T lymphocytes.
- FABP4 is a modulator of DC antigen‐presenting function, which subsequently promotes proper T cell priming.
- Blocking of FABP4 could be a potentially effective approach for therapeutic intervention of T1D.

## 1 Introduction

Type 1 diabetes mellitus (T1D) is an autoimmune‐associated disease characterized by progressive and irreversible β cell destruction by infiltration and attack of immune cells [1, 2]. The initial stage of T1D pathogenesis is triggered by islet resident antigen‐presenting cells, primarily dendritic cells (DCs) and macrophages. DCs can be broadly classified into two main types: plasmacytoid dendritic cells (pDCs) and conventional dendritic cells (cDCs) [3]. cDCs are potent antigen‐presenting cells (APCs) that activate naïve T cells upon maturation following stimulation, while pDCs are known for their ability to secrete large quantities of pro‐inflammatory interferons. Upon activation through nucleic acid‐sensing Toll‐like receptors (TLRs), particularly TLR7 and TLR9, pDCs rapidly produce type I interferon (IFN). Dysregulated production of type I IFN by pDCs has been implicated as a critical mechanism in the pathogenesis of various autoimmune disorders, including T1D [4]. Notably, research by Diana et al. has demonstrated that IFN‐γ‐producing pDCs are recruited to the pancreatic environment, where they initiate diabetogenic T cell responses, thereby contributing to the onset of T1D in NOD mice. Furthermore, studies have indicated that IFN‐α produced by pDCs is essential for the initiation of T1D, and the early‐life depletion of pDCs (between 15 and 25 days of age) significantly delays the onset of diabetes in NOD mice. In humans, a marked increase in the frequency of pDCs has been observed in peripheral blood across all stages of T1D, including at‐risk, newly diagnosed, and established phases. Conversely, cDCs are specialized APCs characterized by their distinctive dendritic morphology and high expression of MHC class II molecules [5]. In murine models, cDCs can be subdivided into two primary subsets: CD8^+^ DCs (which express CD103^+^ in tissue contexts) and CD11b^+^ DCs. Although the precise mechanisms by which CD103^+^ DCs prime β‐cell‐reactive T cells remain to be fully elucidated, emerging evidence suggests that these DCs present islet antigens to T cells in an MHC class II‐dependent manner, following the uptake of secretory granules known as “crinosomes,” which contain insulin and potentially other β‐cell autoantigens with which CD103^+^ DCs are physically associated.

DCs may promote tolerance through various mechanisms, including the generation and maintenance of regulatory T cells (T_Regs_) and the induction of T cell unresponsiveness. However, the robust antigen presentation capabilities of DCs can also facilitate the priming and/or effector differentiation of self‐reactive T cells, potentially due to inappropriate activation signals or intrinsic failures in negative regulatory pathways. Given their pivotal role in the induction and maintenance of self‐tolerance, DCs represent a promising target for therapeutic intervention in autoimmune diseases [6]. Recently, a phase 1/2 clinical study of AVT001, an autologous dendritic cell vaccine, reported improvements in β cell preservation. Nevertheless, the DC vaccine faces limitations, with efficacy being sustainable only for a limited duration. Therefore, a deeper understanding of the underlying causes and mechanisms of DC dysfunction in T1D is essential for advancing the development of effective DC‐based therapies.

Fatty acid binding protein 4 (FABP4, also known as aP2 or A‐FABP), a member of the FABP family, was first identified as a circulating protein secreted from adipose tissue [7]. FABP4 functions as a lipid‐binding chaperone that regulates trafficking and cellular signaling of fatty acids and plays an important role in linking lipid metabolism with innate immunity and inflammation [4]. Recently, studies regarding the relationship between serum FABP4 and T1D have emerged [5, 6, 7, 8, 9, 10, 11]. Serum FABP4 levels were significantly increased in patients with T1D and were closely associated with islet autoantibodies, indicating a potential role of FABP4 in β cell autoimmunity in patients with T1D [10, 11]. Mechanistically, FABP4 induces the infiltration and polarization of macrophages to proinflammatory M1 subtype, thus creating an inflammatory milieu required for the activation of diabetogenic CD8^+^ T lymphocytes and shifting of CD4^+^ helper T lymphocytes toward the Th1 subtypes in islets [10]. During BMDC differentiation in the presence of GM‐CSF and IL‐4, FABP4 expression increased. However, whether FABP4 in DCs participates in the occurrence and development of T1D has not yet been determined [12].

STZ can specifically destroy β cells by binding to the receptor GLUT2 on the pancreas. In contrast to a single high‐dose STZ injection [13, 14, 15, 16], injections of low doses of STZ can trigger an autoimmune process leading to the destruction of the β cells of the pancreatic islets. Herein, we applied this widely used model to study the role of FABP4 in regulating DCs functions that respond to antigens in T1D [17, 18]. We report that FABP4 deficiency decreased T1D incidence and ameliorated hyperglycemia induced by STZ. FABP4 knockout (*FABP4* ^*−/−*^) mice showed lower properties of activated diabetogenic T cells, which is partially due to impaired antigen‐priming functions of DCs. Finally, we studied the activation of the nuclear factor (NF)‐κB pathway in DCs from *FABP4* ^−/−^ and wild type (WT) mice. The results of our study indicate that blocking FABP4 could be a potentially effective approach for the therapeutic intervention of T1D.

## 2 Materials and Methods

### 2.1 Animals

*FABP4* ^*−/−*^ mice in C57BL/6N background were generated using the same procedures as previously described [19]. Age‐matched male *FABP4* ^*−/−*^ and WT mice were used in all the experiments of this study. Animals were allocated to their experimental group according to genotypes. No randomization of mice was used. The investigators were not blinded to the experimental groups. All the mice were housed in specific pathogen‐free conditions with controlled temperature (22°C ± 1°C), humidity (50%–70%), and light (12‐h light/dark cycle) conditions and were housed in independently ventilated cages with free access to water and food unless otherwise indicated. All experimental procedures were approved by the Animal Ethics Committee of The Second Xiangya Hospital, Central South University.

### 2.2 Diabetic Mouse Model

Male C57BL/6N mice received treatment with multiple low doses of streptozotocin (MLD‐STZ). STZ (Sigma‐Aldrich) dissolved in 0.1 M sodium citrate (pH 4.5) was injected intraperitoneally at a dose of 40 mg/kg/day for 5 consecutive days. Non‐fasting glucose levels in venous blood were measured every day starting from the first day of treatment. Blood glucose measurements were performed using a blood glucose test meter (ACCU‐CHEK). Any blood glucose level higher than 13.9 mmol/L (250 mg/dL) was confirmed by another test 24 h later. Overt diabetes was diagnosed by 2 consecutive positive blood glucose tests higher than 13.9 mmol/L.

### 2.3 Intraperitoneal Glucose Tolerance Test (IPGTT)

Mice housed in clean cages were fasted for 16 h before intraperitoneal injection with D‐glucose (1 g/kg). Blood was collected from the tail veins of mice at 0, 10, 15, 30, 45, 60, 75, and 90 min after glucose challenge for the measurement of glucose levels with a blood glucose test meter (ACCU‐CHEK) and strips (Roche Diagnostics) according to the manufacturer's instructions.

### 2.4 Histopathology

Mice were sacrificed by cervical dislocation, and the pancreas was carefully separated, fixed, dehydrated, and embedded in paraffin. The wax block was sliced (5–8 μm) and attached to glass slides. The sections were stained in hematoxylin–eosin solution.

### 2.5 Immunostaining

For immunofluorescence staining, antigen retrieval was first performed on tissue sections in boiled sodium citrate buffer, and then the sections were blocked for 1 h. An appropriate amount of primary antibody (insulin, glucagon) was added to the sections, and they were incubated overnight at 4°C in a wet box, followed by sequential incubation with secondary antibodies at room temperature for 30 min. For the immunohistochemical analysis of pancreatic insulin, the sections were incubated with H_2_O_2_ at room temperature for 10 min after antigen retrieval. After aspirating excess blocking solution, the sections were incubated with primary and secondary antibodies.

### 2.6 Pancreatic Insulin Content

An appropriate amount (approximately 1/4–1/3 volume) of pancreatic tissue was placed into precooled acid‐ethanol extract, cut with clean scissors, and incubated at 4°C overnight. The homogenate was obtained using a magnetic bead homogenizer, placed at −20°C for 3 h, and then centrifuged to extract the supernatant. Tris buffer was added for neutralization, and the insulin level was determined by ELISA (Antibody and Immunoassay Services) and then normalized to the total protein concentration to acquire pancreatic insulin content.

### 2.7 Intracellular Cytokine Assay

Splenic and pancreatic draining lymph node lymphoid cells were stimulated with phorbol myristic acid (100 μg/mL), ionomycin (1 μg/mL) and monensin (1 μL) in cell culture medium at a density of 5 × 10^6^ cells/mL for 5 h at 37°C. An Fc receptor blocker was added to block nonspecific binding. Surface markers were added before fixation and permeabilization, and intracellular cytokine staining was then performed according to the protocol.

### 2.8 Bone Marrow‐Derived Dendritic Cells

Bone marrow cells were flushed from the femur and tibia of either FABP4^−/−^ or WT C57BL/6N mice. Cells were filtered to remove debris, and erythrocytes were lysed and then washed twice with culture medium before culture. Bone marrow cells were counted and resuspended at 1.5 × 10^6^ cells/mL in 3 mL prewarmed culture medium containing 20 ng/mL rmGM‐CSF (Peprotech) and 20 ng/mL rmIL‐4 (Peprotech) and plated on 60 mm culture dishes and incubated at 37°C in 5% CO_2_ humidified air (Day 0). Half of the culture medium was removed and replaced with fresh culture medium every other day. Cells were harvested on Day 7, with approximately 90% of cells being positive for CD11c, which resembles dendritic cells.

### 2.9 Allogeneic Mixed Lymphocyte Reaction (MLR) Assay

Bone marrow‐derived dendritic cells (DCs) harvested from wild‐type (WT) or *FABP4* ^*−/−*^ mice served as the stimulator cells, while splenocytes obtained from non‐obese diabetic (NOD) mice were employed as the responder cells. DCs were meticulously isolated on Day 8 of the culture period following a standardized protocol. Subsequently, the isolated DCs and splenocytes were cocultured in 96‐well U‐bottom plates at an appropriate cell ratio. The coculture was maintained under optimal cell culture conditions for 72 h to allow sufficient interaction between the stimulator and responder cells. After the incubation period, the cells were carefully processed and then subjected to in‐depth analysis using flow cytometry.

### 2.10 Western Blotting

Bone marrow‐derived dendritic cells (BMDC) were stimulated with or without LPS (Sigma‐Aldrich) and harvested at 0, 15, 30, or 60 min post‐stimulation. The total protein content of the cell lysates was determined by a BCA protein assay kit (Pierce) and equal amounts of protein per sample were used for western blotting. Proteins were separated by SDS‐PAGE, transferred to polyvinylidene difluoride membranes, and probed with primary antibodies against mouse ERK (0.348 mg/mL, rabbit monoclonal; Abcam), p‐ERK (0.527 mg/mL, rabbit monoclonal; Abcam), JNK (Abcam) and p‐JNK (Abcam). The intensities of the protein bands were quantified using the ImageJ software.

### 2.11 Statistical Analysis

All experiments were performed at least three times, and the results are presented as the means ± SEM. Statistical analysis and image depiction were accomplished using SPSS 25.0 and GraphPad Prism 5, respectively. Diabetes incidence was compared using the log‐rank (Mantel–Cox) test. The IPGTT results were analyzed with ANOVA, and the remaining data were analyzed using Student's *t*‐test. All statistical tests with *p* < 0.05 were considered significant.

## 3 Results

### 3.1 FABP4 Deficiency Protects Mice From STZ‐Induced Type 1 Diabetes

**FIGURE 1:**
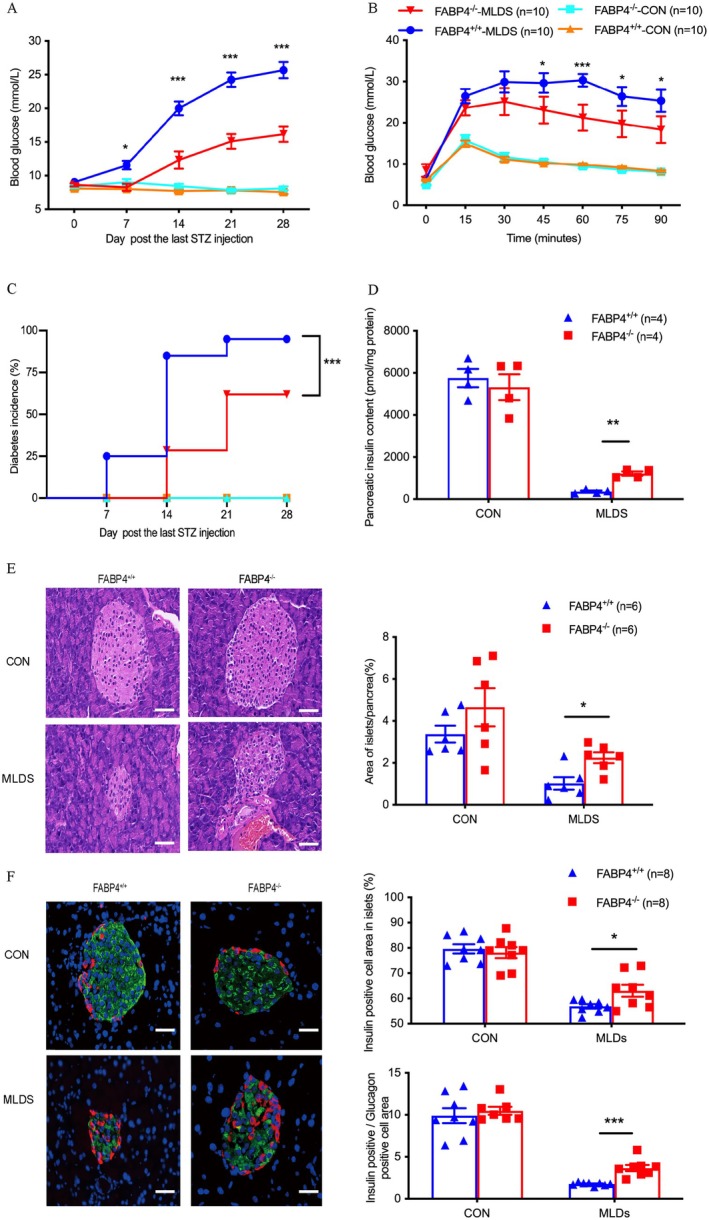
Genetic deletion of FABP4 protects the development of STZ‐induced diabetes. (A) Dynamic blood glucose levels (*n* = 10). (B) Blood levels of glucose during the GTT on day 9 (*n* = 10). (C) Diabetes incidence (*n* = 19–20). (D) Pancreatic insulin content measured by ELISA (*n* = 8). (E) Representative images of H&E analysis for pancreatic sections in pancreas *FABP4* ^*+/+*^ and *FABP4* ^*−/−*^ mice (left panel), and quantification of the area of the islet in each field (right panel) (Magnification 400×, scale bar represents 20 μm) (*n* = 8). (F) Representative images of IHC staining of glucagon (red) and insulin (green) in pancreases of *FABP4* ^*+/+*^ and *FABP4* ^*−/−*^ mice (left panel), and quantification of the percentage of the β cell area in each islet (*n* = 6, right panels). (Magnification 400×, scale bar represents 20 μm). Data are expressed as mean ± SEM. Statistical significance was determined by one‐way analysis of variance or Student's *t*‐test. *p < 0.05, **p < 0.01, ***p < 0.001, *FABP4* ^*+/+*^‐MLDs versus *FABP4* ^*−/−*^‐MLDs. GTT, glucose tolerance test; IHC, immunohistochemistry; MLDs, multiple low‐dosed STZ‐induced diabetes.

To investigate the role of FABP4 in the pathogenesis of T1D, we applied MLD‐STZ to *FABP4* ^−/−^ and WT mice to generate T1D. STZ‐treated *FABP4*‐deficient mice displayed a reduced level of non‐fasting blood glucose elevation compared with STZ‐treated WT mice (Figure 1A), as well as a lower diabetic incidence (Figure 1C). An intraperitoneal glucose tolerance test (IPGTT) was performed on Day 9 after MLDs or vehicle treatment. Compared to STZ‐treated WT mice, the STZ‐treated *FABP4* ^*−/−*^ mice had significantly lower levels of blood glucose after glucose loading (Figure 1B). The insulin content of the pancreas from STZ‐treated *FABP4* ^*−/−*^ mice was obviously higher than that of STZ‐treated WT mice (Figure 1D). H&E staining analysis also showed more islet preservation in STZ‐treated *FABP4* ^*−/−*^ mice than in STZ‐treated WT mice (Figure 1E). Correspondingly, STZ‐treated *FABP4* ^*−/−*^ mice had larger pancreatic β cell areas and smaller pancreatic α cell areas than STZ‐treated WT mice, while there were no differences in pancreatic α cell or β cell areas between vehicle‐treated *FABP4* ^*−/−*^ mice and WT mice (Figure 1F). These results revealed the possible involvement of FABP4 in triggering the STZ‐induced development of insulitis and diabetes in mice.

### 3.2 FABP4 Deficiency Reduces STZ Induction of Diabetogenic T Lymphocytes

**FIGURE 2:**
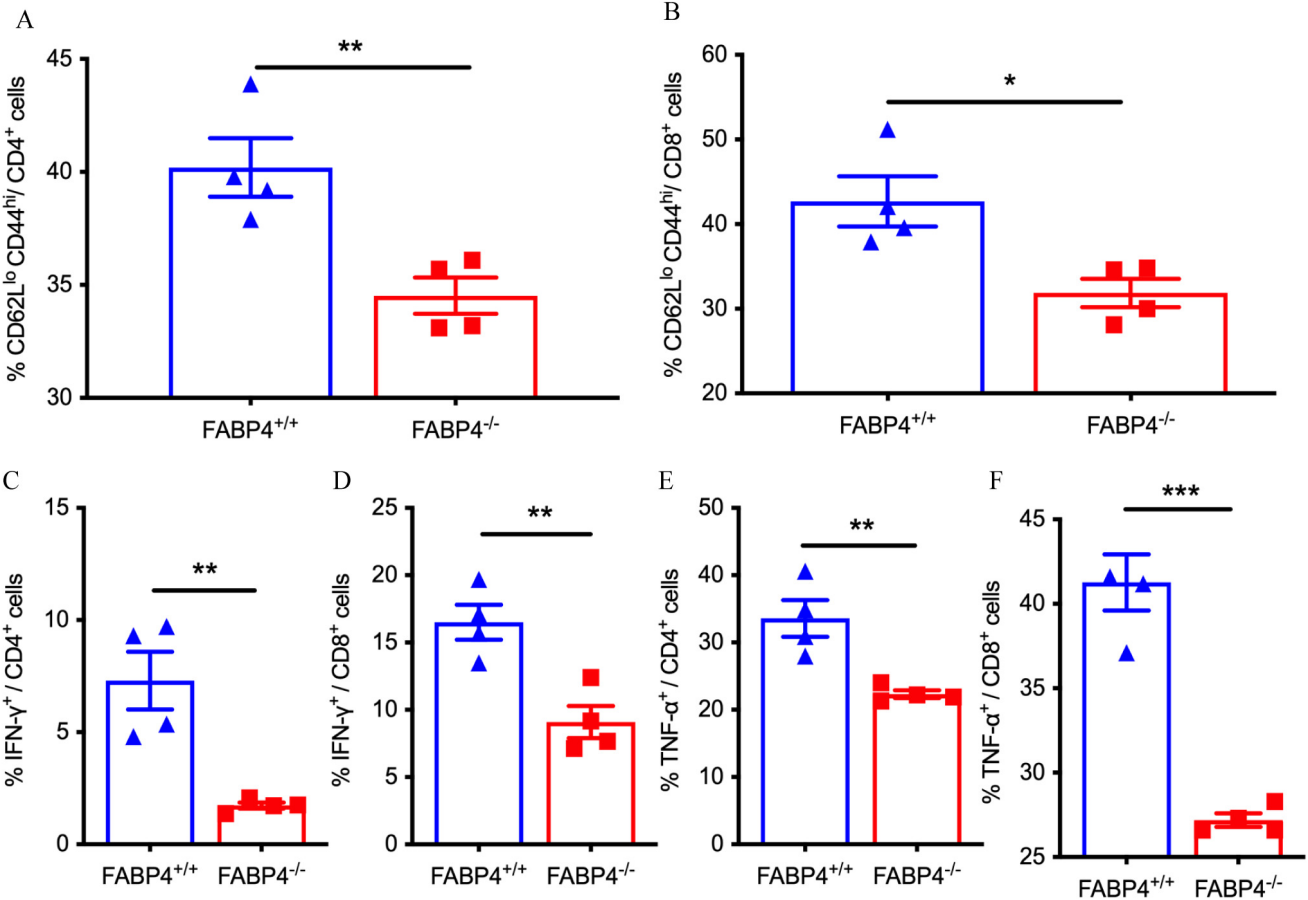
FABP4 deficiency reduces STZ‐induced diabetogenic T cells. (A, B) Summary of the percentages of CD62L^lo^CD44^hi^ effector T cells in *FABP4* ^*−/−*^ and *FABP4* ^*+/+*^ mice (*n* = 4). (C, D) Summary of the percentages of IFN‐γ^+^ T cells in *FABP4* ^*−/−*^ and *FABP4* ^*+/+*^ mice (*n* = 4). (E, F) Summary of the percentages of TNF‐⍺^+^ T cells in *FABP4* ^*−/−*^ mice and *FABP4* ^*+/+*^ mice (*n* = 4). Data are expressed as mean ± SEM of measurements. Statistical significance was determined by one‐way analysis of variance or Student's *t*‐test. *p < 0.05, **p < 0.01, ***p < 0.001, *FABP4* ^*+/+*^‐MLDs versus *FABP4* ^*−/−*^‐MLDs.

The autoimmune destruction of β cells in type 1 diabetes is caused by the activation of diabetogenic T lymphocytes, including CD4^+^ T lymphocytes and CD8^+^ cytotoxic T lymphocytes (CD8^+^ CTLs) [20]. Flow cytometry was applied and showed that splenic T lymphocytes from *FABP4* ^*−/−*^ mice had lower proportions of CD62L^lo^CD44^hi^ activated T lymphocytes than those from WT mice (*p* = 0.010 for CD4^+^ T lymphocytes, *p* = 0.019 for CD8^+^ T lymphocytes) (Figure 2A,B), while the proportions of CD62L^hi^CD44^lo^ naïve CD4^+^ and CD8^+^ T lymphocytes were not significantly different between the two groups of mice (Figure S1A,B) (*p* > 0.050 for both). The levels of an activation inducer molecule, CD69, in *FABP4* ^*−/−*^ mice CD4^+^ and CD8^+^T lymphocytes were not significantly different from those of WT mice (Figure S1C,D) (*p* > 0.050 for both). In addition to surface markers, intracellular cytokines of T lymphocytes of *FABP4* ^*−/−*^ and WT mice were examined. Compared to WT littermates, CD4^+^ and CD8^+^ T lymphocytes of *FABP4* ^*−/−*^ mice produced lower levels of IFN‐γ (*p* = 0.012 for CD4^+^, *p* < 0.001 for CD8^+^) (Figure 2C,D) and TNF‐α (*p* = 0.007 for CD4^+^, *p* = 0.024 for CD8^+^) (Figure 2E,F). In addition to proinflammatory cytokines, the expression of IL‐10, a suppressive factor of inflammation, in CD4^+^ T lymphocytes was not significantly different between *FABP4* ^*−/−*^ and WT mice (Figure S1E) (*p* = 0.998). There was also no difference in the Foxp3^+^ Treg frequency between the two groups of mice (Figure S1F) (*p* = 0.886). These findings suggested that FABP4 mainly promotes the activation of naïve T lymphocytes into effector memory T lymphocytes and the production of proinflammatory cytokines in T lymphocytes, which forms a basis for abnormal T lymphocyte activation and adaptive immune dysfunction in T1D.

### 3.3 Knockout of FABP4 Attenuates the Activation of DCs and the Resulting Induction of Inflammation

**FIGURE 3:**
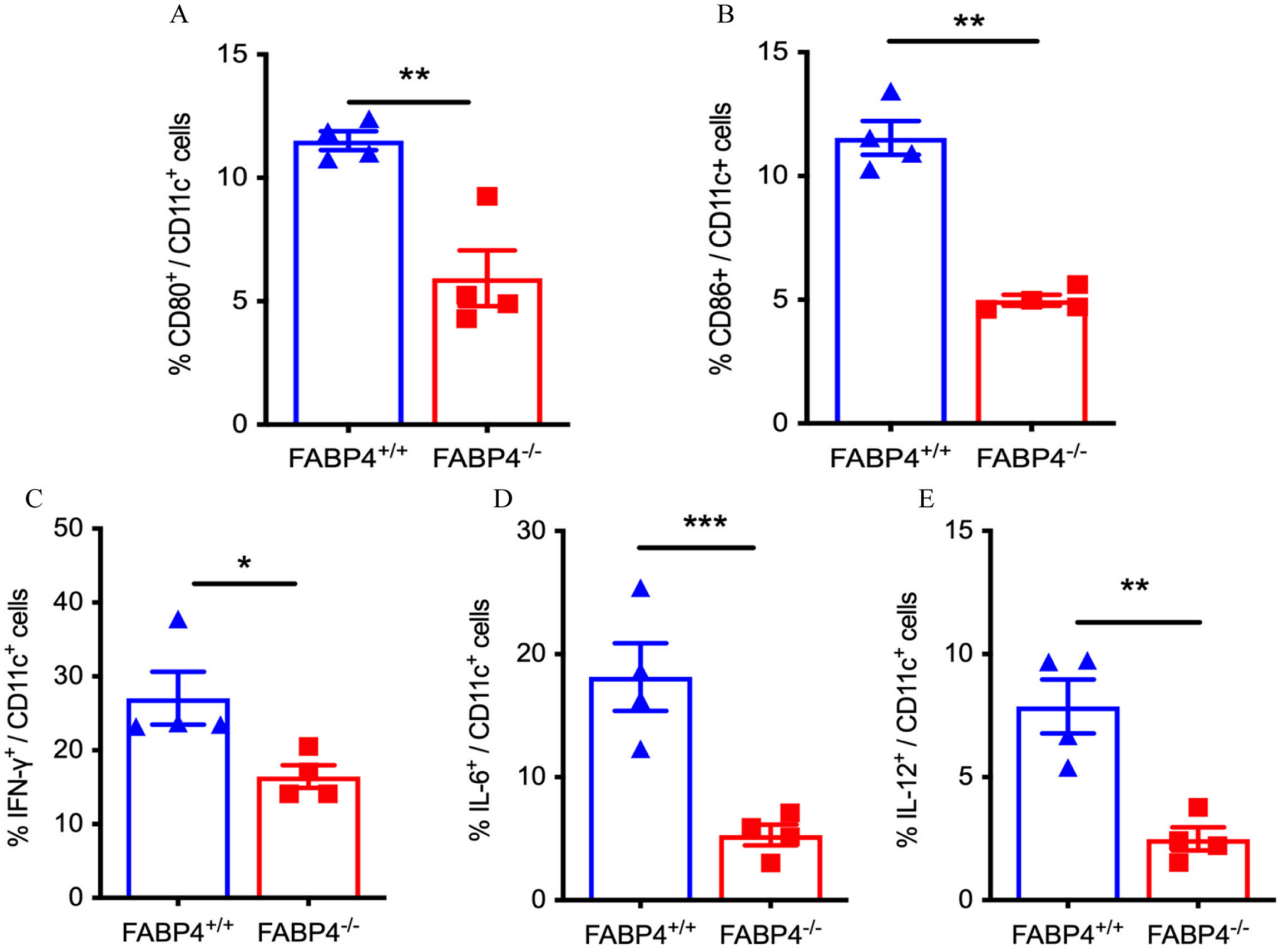
FABP4 deficiency reduces the costimulatory molecules expression of DCs and reduces the inflammatory factors secretion in DCs. (A) Summary of the percentages of CD80^+^ DC cells in *FABP4* ^*−/−*^ and *FABP4* ^*+/+*^ mice (*n* = 4). (B) Summary of the percentages of CD86^+^ DCs in *FABP4* ^*−/−*^ and *FABP4* ^*+/+*^ mice (*n* = 4). (C) Summary of the percentages of IFN‐γ^+^ DC cells in *FABP4* ^*−/−*^ and *FABP4* ^*+/+*^ mice (*n* = 4). (D) Summary of the percentages of IL‐6^+^ DC cells in *FABP4* ^*−/−*^ and *FABP4* ^*+/+*^ mice (*n* = 4). (E) Summary of the percentages of IL‐12^+^ DC cells in *FABP4* ^*−/−*^ and *FABP4* ^*+/+*^ mice (*n* = 4). Data are expressed as mean ± SEM of measurements. Statistical significance was determined by one‐way analysis of variance or Student's *t*‐test. *p < 0.05, **p < 0.01, ***p < 0.001, *FABP4* ^*+/+*^‐MLDs versus *FABP4* ^*−/−*^‐MLDs.

Given that DCs were reported to be the most important APCs contributing to the pathogenesis of T1D, we tested whether lacking FABP4 expression by DCs was necessary for inducing diabetes in STZ‐treated mice [21]. We found that compared to WT mice, CD11c^+^ DCs of *FABP4* ^*−/−*^ mice expressed reduced levels of costimulatory marker CD86 (*p* = 0.003) (Figure 3B) and CD80 (Figure 3A), and there was a trend toward reduced levels of MHCII (*p* = 0.065) (Figure S2A). We further examined intracellular inflammatory cytokine production in the DCs of *FABP4* ^*−/−*^ and WT mice. The data showed that compared to WT mice, CD11c^+^ DCs of *FABP4* ^*−/−*^ mice produced lower levels of IFN‐γ, as well as IL‐6 and IL‐12 (*p* < 0.001 and *p* = 0.001, Figure 3C–E). There was no statistically significant difference in IL‐10 levels between CD11c^+^ DCs of *FABP4* ^*−/−*^ and WT mice (Figure S2B). These results indicated that FABP4 deficiency led to fewer activated DCs and decreased levels of proinflammatory cytokines in DCs, which may protect against the development of autoimmune diabetes.

### 3.4 FABP4 Deficiency Ameliorates the Ability of DCs to Activate CD4 ^+^ T Lymphocytes

**FIGURE 4:**
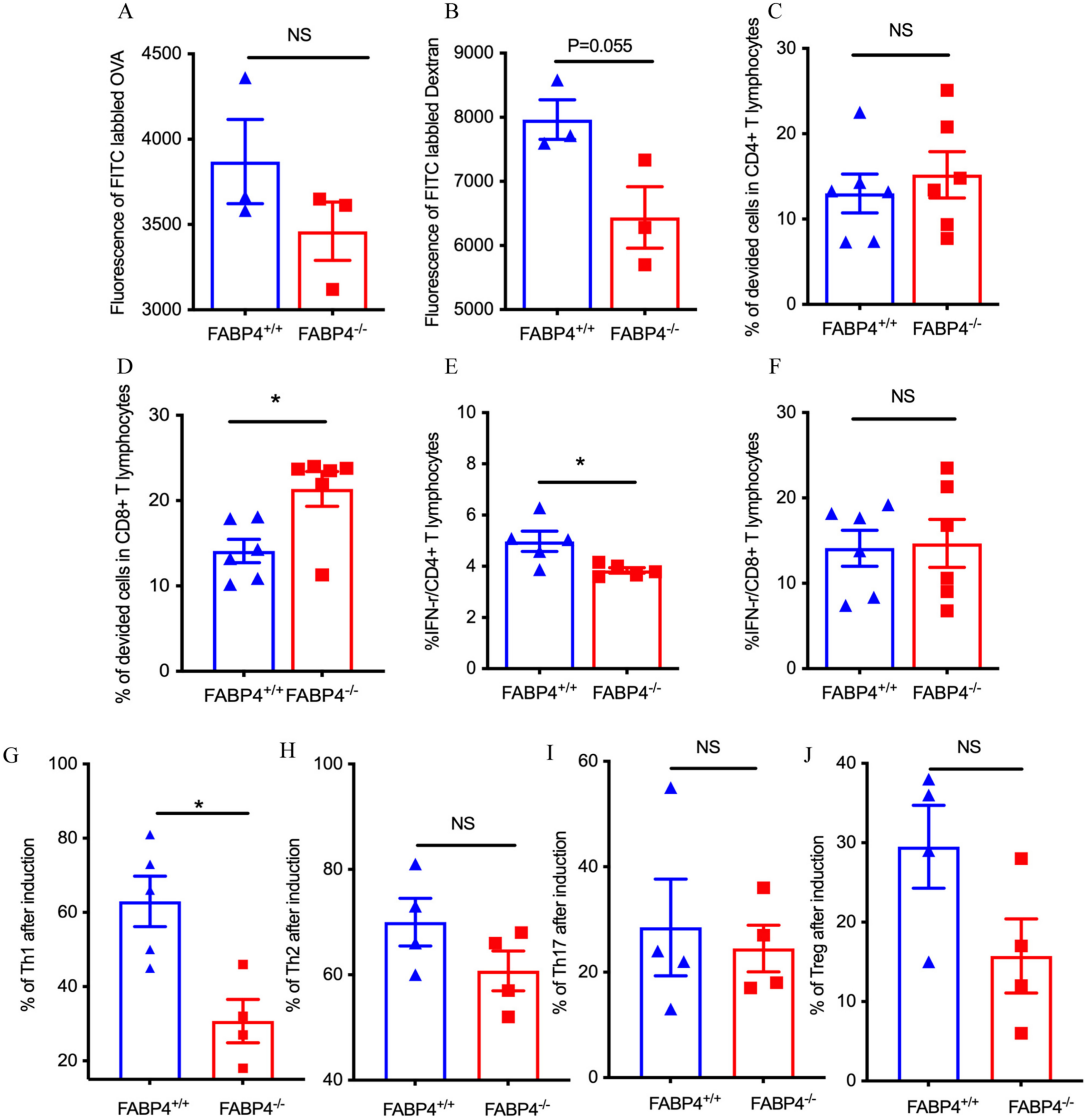
FABP4 deficiency ameliorated the ability of DCs to activate CD4+ T lymphocytes. (A) Summary of the cellular uptake of FITC‐OVA (*n* = 3). (B) Summary of the cellular uptake of FITC‐Dextran (*n* = 3). (C) Summary of the percentages of proliferated CD4^+^ T cells in the coculture system (*n* = 6). (D) Summary of the percentages of proliferated CD8^+^ T cells in the coculture system (*n* = 6). (E) Summary of the percentages of IFN‐γ‐producing CD4^+^ T cells in the coculture system (*n* = 6). (F) Summary of the percentages of IFN‐γ‐producing CD8^+^ T cells in the coculture system (*n* = 6). (G) Frequency of CD4^+^IFN‐γ^+^ Th1 after exposure of naïve CD4^+^ T cells with *FABP4* ^*+/+*^ or *FABP4* ^*−/−*^ BMDCs (*n* = 4–5). (H) Frequency of CD4^+^IL‐4^+^ Th2 after exposure of naïve CD4^+^ T cells with *FABP4* ^*+/+*^ or *FABP4* ^*−/−*^ BMDCs (*n* = 4–5). (I) Frequency of CD4^+^IL17A^+^ Th17 after exposure of naïve CD4^+^ T cells with *FABP4* ^*+/+*^ or *FABP4* ^*−/−*^ BMDCs (*n* = 4–5). (J) Frequency of CD4^+^FOXP3^+^ Tregs after exposure of naïve CD4^+^ T cells with *FABP4* ^*+/+*^ or *FABP4* ^*−/−*^ BMDCs (*n* = 4–5). Data are expressed as mean ± SEM of measurements. Statistical significance was determined by one‐way analysis of variance or Student's *t*‐test.*p < 0.05, NS, not significant.

We further investigated the effect of FABP4 genetic deletion on the functions of DCs [22]. Regarding the ability of DCs to take up antigens, namely endocytosis, distinct mechanisms include phagocytosis, receptor‐mediated endocytosis, or micropinocytosis depending on the nature of the particle to be internalized. The antigen uptake function of DCs was measured by applying FITC fluorescently labeled ovalbumin (OVA) and dextran. We found that DCs of *FABP4* ^*−/−*^ or WT mice showed comparable antigen uptake of OVA (3460.33 ± 170.50 vs. 3869.00 ± 247.08, *p* = 0.245); however, there was a tendency of decreased dextran internalization in DCs from *FABP4* ^*−/−*^ mice compared with those from WT mice (6438.33 ± 477.64 vs. 7963.67 ± 310.54, *p* = 0.055), implicating that FABP4 deficiency has a potential effect on the antigen uptake ability of DCs via a receptor‐mediated pathway (Figure 4A,B).

Another principal function of DCs is thought to be antigen presentation and activation of T lymphocytes [23]. We then tested the requirement for FABP4 in DCs for T lymphocyte activation by mixed lymphocyte reaction assays (MLR). Using DCs from *FABP4* ^*−/−*^ or WT mice as stimulators, the proliferation of NOD splenic cells was measured. CD4^+^ T lymphocyte proliferation was comparable between *FABP4* ^*−/−*^ and WT mice, while CD8^+^ T lymphocyte proliferation was increased in *FABP4* ^*−/−*^ mice (*p* = 0.550 and *p* = 0.049, Figure 4C,D). Interestingly, there was a lower proportion of IFN‐γ‐producing CD4^+^ T lymphocytes stimulated by *FABP4* ^*−/−*^ DCs than by WT DCs, while there was no significant difference in the proportion of IFN‐γ‐producing CD8^+^ T lymphocytes stimulated by *FABP4* ^*−/−*^ DCs and WT DCs (*p* = 0.038 and *p* = 0.878, Figure 4E,F). To further investigate how FABP4 in DCs influences CD4^+^ T lymphocyte subsets, in direct co‐culture experiments, we examined T cells differentiation under T cells‐polarizing conditions. Our data showed *FABP4* ^*−/−*^ DCs significantly suppressed the differentiation of naïve CD4^+^ T cells toward a Th1 (*p* = 0.010, Figure 4G) fate but had no effects on Th2 (*p* = 0.167, Figure 4H), Th17 (*p* = 0.707, Figure 4I) and Treg (*p* = 0.090, Figure 4J). The results suggested a potential role of FABP4 in the pathogenesis of autoimmune diabetes through promotion of the ability of DCs to result in increased production of IFN‐γ in proinflammatory CD4^+^ T lymphocytes.

### 3.5 Absence of FABP4 Leads to Downregulation of Phosphorylated ERK and JNK Pathways in DCs

**FIGURE 5:**
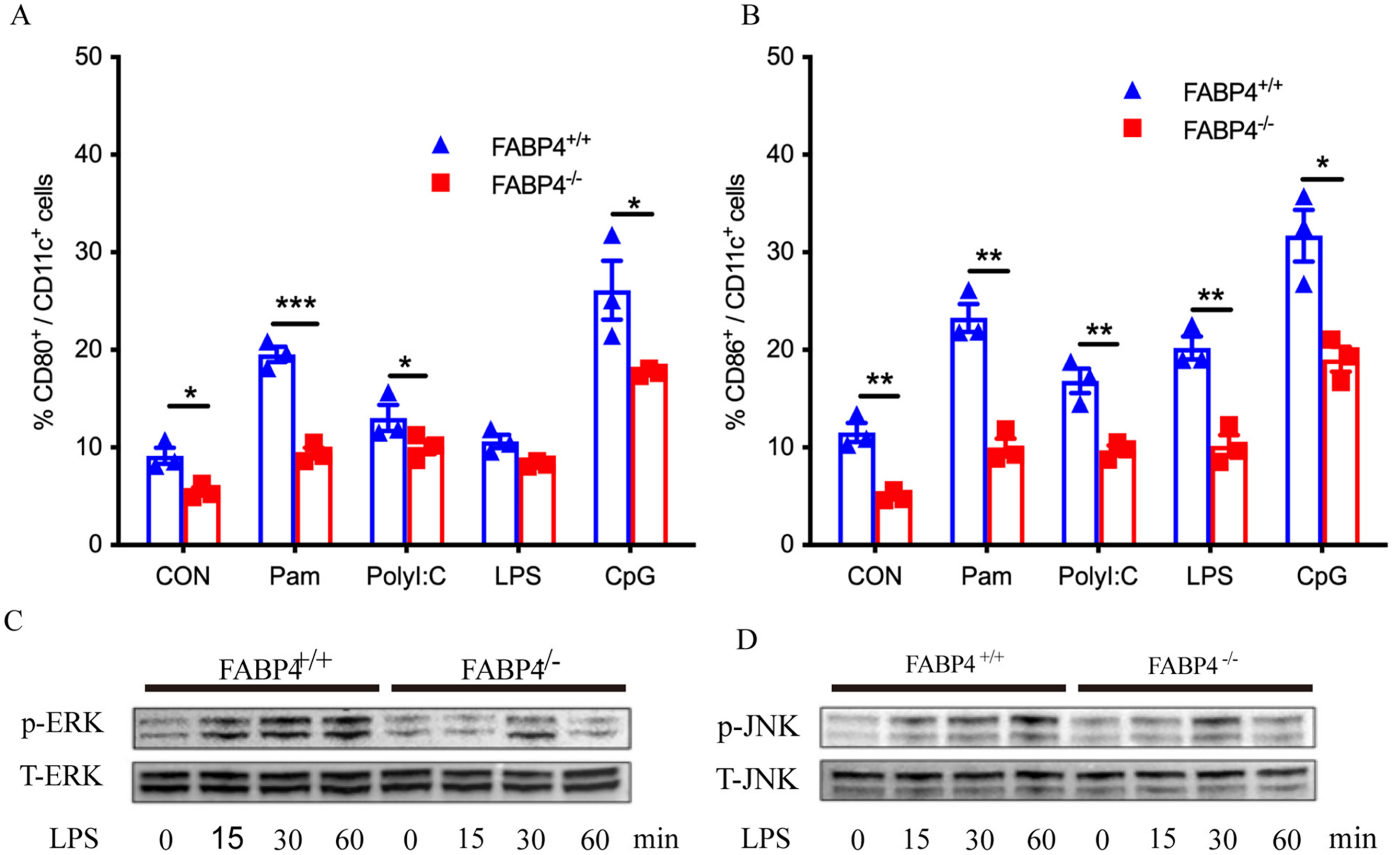
Potential mechanism of impaired function of DCs in the absence of FABP4. (A) Summary of the percentages of CD80^+^ DC cells in BMDCs stimulated with TLR agonists (*n* = 3). (B) Summary of the percentages of CD86^+^ DC cells in BMDCs stimulated with TLR agonists (*n* = 3). (C, D) Western blot for phosphorylation of MAPKs. BMDCs from *FABP4* ^*−/−*^ and *FABP4* ^*+/+*^ mice were stimulated by LPS for 15, 30, 60, or 90 min. Data are expressed as mean ± SEM of measurements. Statistical significance was determined by one‐way analysis of variance or Student's *t*‐test. *p < 0.05, **p < 0.01, ***p < 0.001.

Among the most interesting functional DC abnormalities, implicated both in the mice model and in T1D, is the dysregulated or enhanced nuclear factor (NF)‐κB pathway [24]. As FABP4 works as a downstream effector of Toll‐like receptors (TLRs) in immune cells, we first examined whether the absence of FABP4 could affect the activation of DCs by TLR ligation [25]. Various TLR agonists, including Pam3CSK4 (TLR2), PolyI:C (TLR3), LPS (TLR4) and CpG (TLR9), were administered to splenic DCs. The results of flow cytometry demonstrated lower expression levels of the costimulatory molecules CD80 and CD86 in BMDCs from *FABP4* ^*−/−*^ mice than in those from WT littermates in response to Pam3CSK4, PolyI:C, LPS, and CPG (Figure 5A,B), but the expression of the MHCII molecule in CD11c^+^ DCs was not different between the two groups of mice, regardless of TLR ligation (Figure S3A).

It is now well established that TLRs activate the NF‐κB and MAP kinase pathways to drive immune responses [26]. Furthermore, studies demonstrated that FABP4 regulates TLR‐stimulated NF‐κB and MAP kinase pathways in macrophages [27]. Studies based on the role of FABP4 in DCs are much less common; thus far, FABP4 has been shown to activate the IKK‐NF‐κB pathway in DCs [12]. Hence, to determine whether the MAPK signaling pathway was affected in DCs from *FABP4* ^*−/−*^ mice in response to TLR stimulation, we examined the activation of the MAPK/ERK and MAPK/JNK pathways in BMDCs from *FABP4* ^*−/−*^ mice and WT mice after LPS stimulation. After LPS stimulation, the phosphorylated ERK protein levels of the two different genotypes increased compared to those without LPS stimulation. However, BMDCs of *FABP4* ^*−/−*^ mice displayed a peak expression of p‐ERK at 30 min and were significantly hydrolyzed at 60 min, while the BMDCs of WT littermates showed an obvious increase in the p‐ERK protein at 15 min and persisted at 60 min (Figure 5C). Similarly, changes were also seen in the alterations of the JNK protein after LPS stimulation. DCs of the knockout mice had a delayed peak expression and an earlier hydrolysis when compared with WT mice; the latter had a significant increase in p‐JNK at 15 min and persisted at 60 min (Figure 5D). Thus, the potential mechanism of the reduced activation and impaired function of DCs with FABP4 deficiency is attributed to the downregulation of the phosphorylated ERK and JNK pathways.

## 4 Discussion

Targeting DCs is considered an appealing strategy to modulate autoimmune disorders in an antigen‐specific manner and to intervene in the pathogenesis of T1D; however, the molecular mechanism by which DCs regulate T lymphocytes in T1D has not been fully elucidated, and potential drug targets for DCs are scarce. Thus, identifying the factors that regulate the function of DCs in regard to T lymphocytes is crucial for the discovery of novel interventions for T lymphocyte‐mediated autoimmune diabetes. Here, we demonstrated that FABP4 deficiency ameliorated STZ‐induced diabetes onset, insulitis, activation, and imbalance of T lymphocytes in C57BL/6N mice. FABP4 deficiency impaired the activation of DCs, attenuated the activation of MAP kinase pathways, and reduced the production of inflammatory cytokines in DCs, resulting in ameliorated ability of DCs to activate the proliferation and the expression of inflammatory cytokines of CD4^+^ T lymphocytes, suggesting a weakened role of DCs in contributing to T lymphocyte‐triggered insulitis. Our present study provides new evidence for developing FABP4 as a potential prevention and therapeutic target for T1D.

The inflammatory activities of FABP4 in different immune cells has been reported previously in the context of obesity, atherosclerosis, nonalcoholic fatty liver disease and rheumatoid arthritis (RA) [7, 28, 29, 30]. FABP4 in macrophages is a well‐known mediator that enhances proinflammatory M1 subtype polarization thus activating diabetogenic CD8^+^ T cells and shifting CD4^+^ T cells toward Th1 subtypes [10]. Genetic ablation or pharmacological inhibition of FABP4 attenuates M1 macrophages polarization. FABP4 in islets tissue‐resident memory T (T_RM_) cells potentiates the survival and alarming function of T_RM_ cells by promoting fatty acid utilization and CXCL10 secretion [31]. In DCs, FABP4 regulates the secretion of selective cytokines such as IL‐12 and TNF thereby modulating the T cell priming [12]. Moreover, DCs deficient in FABP4 were found to be poor producers of proinflammatory cytokines, and Ag presentation by *FABP4* ^*−/−*^ DCs did not promote proinflammatory T‐cell responses in experimental autoimmune encephalomyelitis (EAE) [32, 33]. Previous studies have demonstrated that FABP4 exacerbates the onset and progression of T1D by promoting the polarization of M1 macrophages in NOD mouse models [10]. Additionally, the expression of cytokines, such as IL‐12 and TNF, is significantly impaired in BMDCs from FABP4 knockout mice. In our study, we utilized an STZ‐induced T1D mouse model to further validate the protective role of FABP4 gene knockout in the progression of T1D [12]. Furthermore, there was a lower proportion of activated cells (CD62L^lo^CD44^hi^) among both CD4^+^ and CD8^+^ T lymphocytes in FABP4 knockout mouse spleens and decreased IFN‐γ‐ and TNF‐α‐producing T lymphocytes, which may be the reason why FABP4 deficiency protected mice from STZ‐induced T1D development. We hypothesized that DCs, as the major innate immune cells, activate T lymphocytes and that FABP4 expressed by DCs may play a pivotal role in the ability of DCs to activate diabetic T lymphocytes.

DCs recognize antigens through pattern recognition receptors or TLRs, causing consequent phosphorylation of various intracellular kinases, including IkB kinase, ERKs (ERK1/2), p38 MAPK, and JNK1/2 [16, 17]. NF‐kB is an important transcription factor regulating innate and adaptive immunity, and it induces proinflammatory cytokines in myeloid populations [34, 35]. DC maturation results in the production of functionally different effector DC subsets that release polarizing signals (the most important of which are cytokines), which promote the development of Th1, Th2, or Th17 cell responses [12, 18]. Inhibition of the IKK/NF‐κB and JNK signaling pathways in DCs inhibited the differentiation of Th17 cells in vitro, ameliorating the development of EAE [36]. In the development of autoimmune uveitis, enhanced activities of DCs, depending on JNK, ERK, and p38 activation, significantly promoted Th17 responses, while inhibition of ERK completely abolished the Th17 responses induced by activated DCs [37]. Our data showed that FABP4‐deficient DCs had a delayed and weakened response to TLR ligand stimulation. Thus, we speculated that the FABP4 participated in T1D development partially by increasing phosphorylation of ERK and JNK in DCs, resulting in the upregulation of MHCII molecules and costimulatory molecules, including CD80 and CD86, and overactivation of diabetogenic T cells.

Although the effects of inhibition of FABP4 in human T1D remain unclear, findings from the present study and other groups suggest that FABP4 may be a promising therapeutic target for T1D. Hormonal FABP4 forms a functional hormone complex Fabkin with ADK and NDPK, and directly alters β cell calcium dynamics and promotes β cell death [11]. Macrophage‐derived FABP4 enhanced proinflammatory M1 polarization of macrophages, inducing insulitis [10]. In our study, we clarified how FABP4 in DCs activates diabetogenic T lymphocytes, providing evidence for the mechanism by which FABP4 in DCs is involved in disease pathogenesis. Taken together, we have sufficient evidence showing that FABP4 is involved in the development of T1D through intracellular and extracellular mechanisms in different cells, indicating that FABP4 is an interventional target for T1D from multiple dimensions. Moreover, as FABP4 shows restrictive expression patterns, targeting FABP4 for T1D is relatively safe. The pharmacological inhibition of FABP4 by BMS309403 shows beneficial effects in cardiometabolic diseases [38]. The application of FABP4 neutralizing antibodies exhibited therapeutic potential in obesity‐related impairments in glucose metabolism and systemic inflammation [39]. Further studies are needed to explore the translational potential of targeting FABP4.

Although the present study provides substantial evidence supporting the notion that FABP4 potentiates DCs‐initiated autoimmune pathogenesis in T1D, mediating the crosstalk between innate immunity and adaptive immunity in type 1 diabetes, there are still several limitations: (1) Given it is difficult to pick islets in the STZ‐induced T1D mouse model, we have not reported the direct immune cell infiltration in islets; (2) A previous study has reported DC from spleen or BM cultures show a comparative ability to stimulate T cell responses, but it may not mimic the behavior of the key CD103^+^ subset [40]; (3) Our previous study reported that both macrophages and DCs are major sources of FABP4 in the early stages of insulitis, and adaptive transfer experiments are worthy of excluding effects of FABP4 from other cell types [10]. At all events, we expect that the present research will inspire a better understanding of FABP4 to be exploited for T1D therapy.

In summary, our results uncover that FABP4 plays a pathogenic role in T1D development, which is most likely mediated by APCs. This study reveals a novel role of FABP4 in autoimmune diabetes and its potential mechanism. Understanding the potential mechanism by which FABP4 and the related signaling pathways are involved in T1D could open a new view of the pathogenesis of the disease. Finally, comprehensive knowledge gained from clinical and animal perspectives may make FABP4 a new target for the treatment of T1D in the future.

## Author Contributions

Yang Xiao, Zhiguang Zhou, and Aimin Xu designed and supervised the study. Hailan Zou, Xiaoyu Xiao, Jingyi Hu, Yanfei Wang, and Rong Zhang conducted the experiments. Hailan Zou and Xiaoyu Xiao analyzed the data. Hailan Zou wrote the manuscript. Lingxiang Xie and Jingyi Hu helped in the manuscript revision. All authors approved the manuscript.

## Conflicts of Interest

Editorial Board Members (with no EIC or AE role) are co‐authors. Zhiguang Zhou and Aimin Xu are Editorial Board members of Journal of Diabetes and co‐authors of this article. To minimize bias, they were excluded from all editorial decision‐making related to the acceptance of this article for publication.

## Supporting information


**Data S1.** Supporting information.

## Data Availability

All data generated or analyzed during this study will be available by contacting the corresponding author.
