# Supplementary material for: Fatty Acid Binding Protein 4 Regulates the Antigen‐Presenting Function of Dendritic Cells Resulting in T Cell Priming in Streptozotocin‐Induced Type 1 Diabetes Mice
Source: J Diabetes. 2025 Jul 26;17(7):e70123. doi: 10.1111/1753-0407.70123 (PMC12304523; doi:10.1111/1753-0407.70123)
Supplement: Supplementary file 1 — Data S1. Supporting information. [file JDB-17-e70123-s001.pdf]

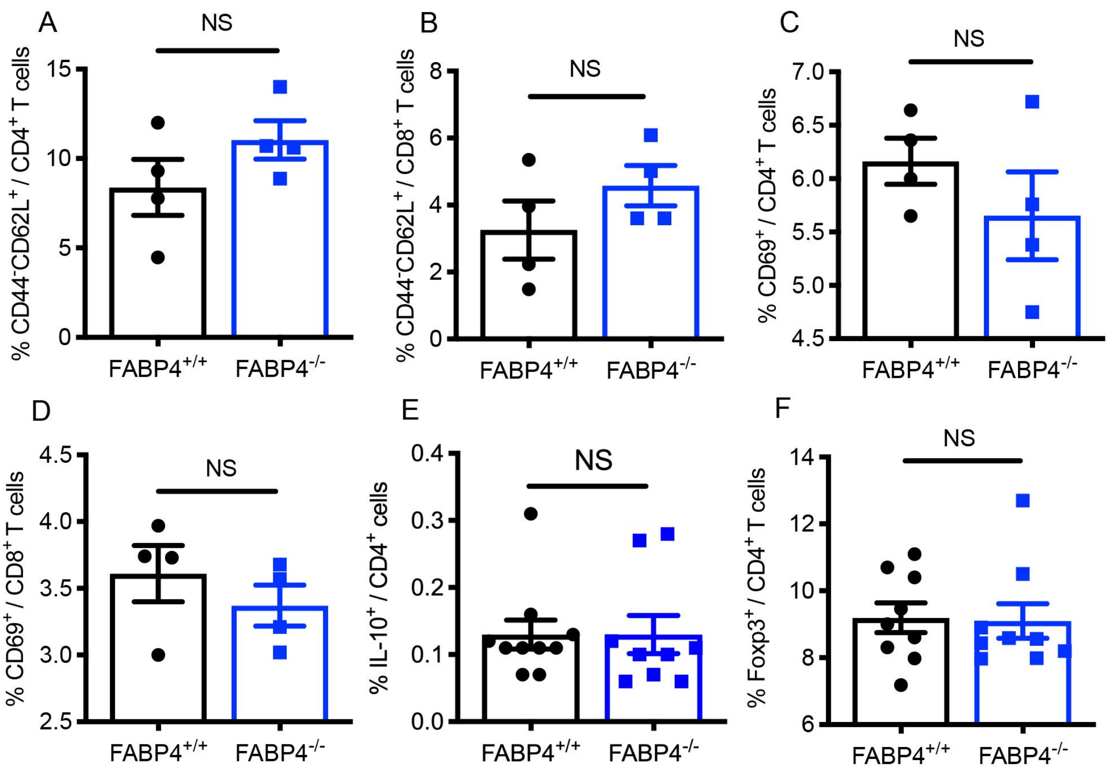

Figure S1. (A, B) Summary of the percentages of $CD62L^{hi}CD44^{lo}$ naive T cells in $FABP4^{-/-}$ and $FABP4^{+/+}$ mice (n=4). (C, D) Summary of the percentages of $CD69^{+}$ T cells in $FABP4^{-/-}$ and $FABP4^{+/+}$ mice (n=4). (E, F) Summary of the percentages of $IL\text{-}10^{+}$ T cells and $Foxp3^{+}$ Treg cells in $FABP4^{-/-}$ and $FABP4^{+/+}$ mice (n=10). Data are expressed as mean ± SEM of measurements. Statistical significance was determined by one-way analysis of variance or Student's t-test. *, $p < 0.05$; ***, $p < 0.001$; NS, not significant, $FABP4^{+/+}$-MLDs vs $FABP4^{-/-}$-MLDs.

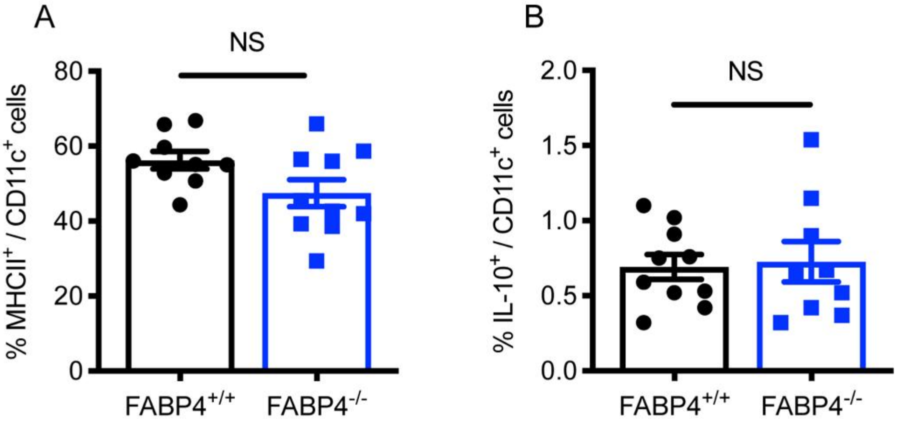

Figure S2. (A) Summary of the percentages of MHC Ⅱ ⁺ DCs in $FABP4^{-/-}$ and $FABP4^{+/+}$ mice (n=10). (B) Summary of the percentages of $IL\text{-}10^{+}$ DCs in $FABP4^{-/-}$ and $FABP4^{+/+}$ mice (n=10). Data are expressed as mean ± SEM of measurements. Statistical significance was determined by one-way analysis of variance or Student's t test. *, $p < 0.05$; ***, $p < 0.001$; NS, not significant, $FABP4^{+/+}$-MLDs vs $FABP4^{-/-}$-MLDs.

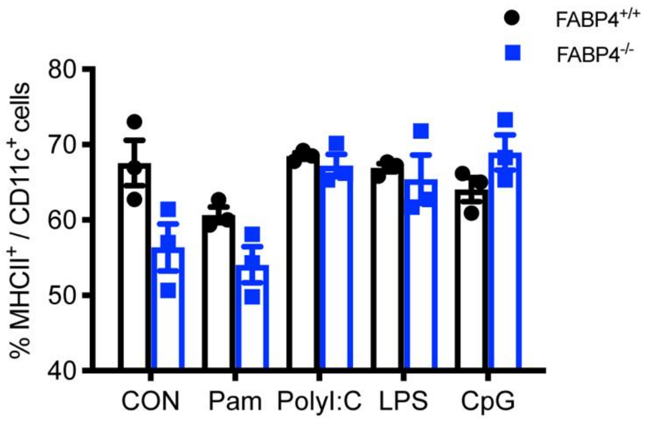

Figure S3. Summary of the percentages of CD86^{+}DC cells in FABP4$^{-/-}$ and FABP4$^{+/+}$ mice (n=3). Data are expressed as mean ± SEM of measurements. Statistical significance was determined by one-way analysis of variance or Student's t-test. *, $p < 0.05$; ***, $p < 0.001$; NS, not significant, FABP4$^{+/+}$-MLDs vs FABP4$^{-/-}$-MLDs.
